# Adverse Reactions after the Third Dose of the BNT162b2 mRNA COVID-19 Vaccine among Medical School Residents in a Regional Reference University Hospital in Italy

**DOI:** 10.3390/vaccines10111779

**Published:** 2022-10-22

**Authors:** Alborz Rahmani, Guglielmo Dini, Alfredo Montecucco, Andrea Orsi, Laura Sticchi, Alexander Domnich, Bianca Bruzzone, Luca Pellegrini, Alessia Manca, Matilde Ogliastro, Bruno Kusznir Vitturi, Sonia Zacconi, Nicoletta Debarbieri, Giancarlo Icardi, Paolo Durando

**Affiliations:** 1 Department of Health Sciences, University of Genoa, 16132 Genoa, Italy; 2 Occupational Medicine Unit, IRCCS Ospedale Policlinico San Martino, 16132 Genoa, Italy; 3 Hygiene Unit, IRCCS Ospedale Policlinico San Martino, 16132 Genoa, Italy

**Keywords:** COVID-19, mRNA vaccine, booster dose, occupational health, healthcare workers, reactogenicity

## Abstract

The recent emergence of new variants of concern (VOCs) of SARS-CoV-2 and the uncertain duration of protection provided by the primary immunization cycle have highlighted the need for COVID-19 booster vaccinations. However, only a few studies have assessed the safety and reactogenicity profile of mRNA booster doses. Therefore, we conducted an online survey with the aim of assessing the adverse reaction profile in the 7 days following a third dose of the BNT162b2 vaccine in a population of resident physicians who had already been investigated after the primary vaccination. Among the 512 resident physicians (female = 53.2%, mean age = 29.8 years) invited to participate in the survey, 222 completed the survey (56.5% female, mean age of 29.9 years), with an average time from second to third dose of 8.6 months. The most common adverse reactions were local pain (88.3%), fatigue (58.1%), muscle/joint pain (44.1%), and headache (38.3%), all subsiding in 48–72 h. While the local reaction rate was similar to that following the first two doses, the systemic reactions were considerably less common and milder compared to the second vaccination. Nonetheless, over one third (36.1%) of participants reported interference with their normal activities. These results complement our previous findings and could aid occupational and public health professionals in the counselling of vaccinees.

## 1. Introduction

Worldwide COVID-19 vaccination campaigns have been shown to be highly effective mitigation tools in reducing the risk of symptomatic SARS-CoV-2 infection and the severity of the disease [1]. However, with the emergence of several new variants of concern (VOCs) of SARS-CoV-2 causing breakthrough infections among vaccinated people, particularly following the global spread of the delta variant in May 2021 [2], uncertainty about the duration and efficacy of protection provided by the primary cycle immunization over time has surfaced. Preliminary real-world research, particularly concerning mRNA vaccinations, showing waning humoral immunity and circulation of SARS-CoV-2 variants capable of eluding neutralization induced by prior vaccination, suggests the need for a third vaccine dose used as a booster, in order to enhance the immune response and reduce the risk of infection [3]. As a result, many developed countries expanded eligibility for a single booster dose of mRNA vaccines among immunocompetent individuals after at least 6 months from the completion of the first cycle [4,5]. In Italy, the Ministry of Health updated its recommendation for healthcare workers, as well as other at-risk categories, to undergo a booster dose administration from September 2021, becoming mandatory from 15 December 2021 [6]. However, only few studies have assessed the safety profile and reactogenicity of mRNA booster doses [7], with the BNT162b2 booster dose clinical trial results published at the end of March 2022 [8]. Our research group has previously published a study on the real-world reactogenicity profile reported by resident medical doctors after the primary immunization cycle of two 30 μg doses of the BNT162b2 vaccine [9], demonstrating a significant increase in systemic adverse reactions after the second vaccination compared to the first dose, impacting in several instances on regular daily activities. Therefore, we conceived the present study with the aim of assessing the reactogenicity following a third dose of COVID-19 mRNA vaccine in the same young working-age population as the original study, and in order to evaluate differences between primary and booster vaccination. The findings could improve the understanding of expected adverse reactions and provide additional relevant information for occupational and public health professionals who are at the forefront of the implementation of this protective and preventive measure. Indeed, up-to-date monitoring of the reactogenicity of COVID-19 vaccines in real-world settings, particularly in the working-age population, among whom these events could interfere with regular work activity, could aid healthcare professionals in setting expectations and potentially improving compliance and acceptance of immunization recommendations.

## 2. Materials and Methods

A cross-sectional observational study was performed using a self-administered electronic questionnaire (Supplementary Material) and distributed with the open-source online software LimeSurvey (Version 4.3.28., LimeSurvey GmbH, Hamburg, Germany). In consideration of the non-concurrent administration of the survey with the vaccination, we created a shortened version of the questionnaire (25 questions) compared to the one used in our previous study following the first two doses of mRNA vaccination (81 questions). The investigated items focused on the same areas as the original study (i.e., type of adverse reaction; grading of intensity as defined in Appendix A; duration of event; and use of non-steroidal anti-inflammatory drugs–NSAIDs) [9]. The survey included questions on demographics (age, sex) and different solicited local or systemic adverse events occurring in the seven days following the administration of the third dose of the mRNA vaccine. The booster vaccination consisted of a single intramuscular injection in the deltoid muscle of 0.3 mL of BNT162b2 vaccine (developed and produced by Pfizer/BioNTech, New York City, NY, USA/Mainz, Germany) after at least 6 months following the primary cycle completion, as recommended by the manufacturer and the Ministry of Health at the time of administration. The study was carried out between 14 December 2021, and 22 February 2022, and it involved the resident doctors of the University of Genoa employed at Policlinic Hospital San Martino of Genoa, Italy, the regional tertiary adult acute care reference hospital, who underwent COVID-19 immunization and were invited to participate in the first study. Vaccinated resident doctors were contacted by email and were invited to participate via a link to the survey, with a unique access token. Participation was voluntary and every participant was required to agree with the LimeSurvey privacy policy. To enter the survey, participants had to consent to an online form with specific information about the purpose and description of the study. Partially completed surveys were discarded, and only fully completed surveys were included in the final analysis. To account for the possible effects of the primary immunization cycle on the adverse events following the booster injection, differences in adverse reactions between doses were evaluated solely using data from subjects that completed surveys after each dose of the vaccination. All data were extracted and exported to Microsoft Excel (Microsoft, Redmond, WA, USA) and then analyzed using the Statistical Package for the Social Sciences (SPSS, IBM Corp., Armonk, NY, USA) software, version 26. Nominal and ordinal categorical variables were summarized and described as frequency and percentages. The Clopper–Pearson Exact method was used to calculate confidence intervals (CIs) for proportions. The Cochran’s Q test was used to compare paired proportions for non-parametric nominal data following each of the three vaccine doses; when statistical significance was found, the McNemar’s test was used to compare paired proportions between each of the two paired groups. The χ2 test and Fisher’s exact test were used for the univariate analysis of the association between sample characteristics and the frequency of the reported adverse reactions. Statistical significance was defined as a two-tailed p < 0.05. The study was managed by the Ethics Committee of the Liguria Region (administrative reference number: 449/2022 ID 12601). All activities were performed in compliance with the Declaration of Helsinki. The data were anonymized before the analysis. Personal information regarding all subjects included in the investigation was protected according to Italian law.

## 3. Results

The entirety of the invited population of the original study (512 resident doctors, female = 53.2%, mean age = 29.8 years, SD 2.7) received a third dose of COVID-19 vaccination and were contacted to participate in the study. Among these, 294 entered the survey (an acceptance rate of 57.4%), of which 222 resident doctors fully completed the survey (56.5% female, mean age of 29.9 years, SD 3.3). Within this sample, the third dose vaccination was performed an average of 4.1 weeks prior (SD 2.9), and the average time passed between the second and third dose administration was 8.6 months (SD 1.3). Concerning local adverse reactions, the most frequently reported were, in order, pain at the injection site (88.3%), swelling (32.0%), and redness (9.0%). Stratification by event severity can be found in Table 1.

Regarding systemic adverse reactions, the most commonly reported events following the third dose were fatigue (58.1%), headache (38.3%), muscle/joint pain (44.1%), lymph node enlargement (28.4%), and fever (27.9%). Details on the stratification by event severity can be found in Table 2.

The three subjects that reported neurological signs and symptoms were contacted by the OHS medical team in order to gather a more detailed account of the reported events. Two could be reached:

- The first case (a female, 29 years old, with a history of migraine with a frequency of about one episode per month) reported two episodes of migraine with aura in the 72 h following the booster vaccination, in the absence of fever, for which she sought specialist attention from a neurologist. Following treatment with triptans, the events subsided, and she did not suffer from further episodes;
- The second case (male, 29 years old, no relevant medical history) reported heavy shaking in the torso, in the absence of fever, lasting the first night after the vaccination.

The median duration of adverse reactions for all local and systemic reactions after the booster vaccination ranged between 1 and 2 days, the longest being lymph node enlargement, lasting a median of 4 days (IQR 3–5).

Subjects presenting at least one severe event that prevented regular daily activities were 29 after the booster dose (13.1%).

Excluding the severe cases, 23.0% of residents showed multiple moderate reactions that interfered with regular activities following the third dose of vaccination, as shown below (Table 3).

During the univariate analysis, significant associations were found between female gender and reporting of headache (χ^2^ = 6.473, *p* = 0.011; OR = 2.07, 95%CI 1.18–3.63); lymph node enlargement (χ^2^ = 3.838, *p* = 0.050; OR = 1.83, 95%CI 1.00–3.37); and use of NSAIDs and anti-pyrectics (χ^2^ = 5.386, *p* = 0.020; OR = 1.88, 95%CI 1.10–3.22). Time between primary vaccination cycle completion and booster administration was positively associated (per 1 month increase) with fatigue (OR = 1.54, 95%CI 1.20–1.99); presenting chills (OR = 1.40, 95%CI 1.04–1.90); and headache (OR = 1.42, 95%CI 1.09–1.84). Use of fever lowering drugs was negatively associated with age (per 1 year increase) (OR = 0.88, 95%CI 0.79–0.97) but positively associated with time since primary cycle completion (per 1 month increase) (OR = 1.51, 95%CI 1.18–1.95).

Considering the sample that completed the survey after each of the three doses of vaccination (N = 111; female = 55.0%; mean age = 29.8 years SD 2.9), performing the booster administration after an average of 8.6 months (SD 1.2) from the primary vaccine schedule, and who completed the survey an average of 3.9 weeks (SD 1.7) after the third dose, no significant difference was found concerning the local adverse reaction frequency between the third and the first two vaccine doses.

Concerning systemic adverse reactions, however, significant differences were present for most symptoms, as well as for anti-inflammatory/antipyretic medication use, as detailed in Table 4.

## 4. Discussion and Conclusions

The present study supplements the findings from our previous research on the reactogenicity of the BNT162b2 mRNA COVID-19 vaccine [9], showing that, for most systemic adverse reactions, reporting after the booster dose was significantly less frequent and milder compared to the second dose. In fact, concerning particular reactions such as headaches and gastrointestinal events, the proportion was as low as after the first vaccination. This is in line with previously published real-world studies [10,11,12,13] as well as clinical trial results [8]. Interestingly, however, reporting of lymph node enlargement and fever remained elevated at the levels following the second dose of vaccination. This was also reflected by the corresponding use of antipyretic medication. These events may be explained by the employment of the secondary immune response, manifesting a prevalent activation of memory cells in lymphoid organs [14,15], stimulated by the booster dose. Moreover, while the simultaneous presence of moderate events interfering with regular activities was reduced, severe events that prevent regular daily activities were not less prevalent compared to the second vaccination. Furthermore, rare adverse reactions, such as neurological symptoms, although transient, were once more reported after this vaccination, indicating that adverse reactions might be expected. The effect of gender on the reactogenicity profile found after the primary immunization cycle was also confirmed following the third dose. This association could be due to several factors, including hormonal [16], genetic [17], and behavioral [18]. Interestingly, a positive association was also found between the time from the primary cycle to the third dose and several systemic reactions and anti-inflammatory or anti-pyretic medicine use. This could be in part indicative of a higher immunogenic effect due to a greater degree of waning immunity prior to the booster administration. However, the association between reactogenicity and immunogenicity of this vaccine is still under study [19].

Overall, these findings suggest that, although the booster dose appears to cause fewer reactions, accurate surveillance programs of adverse events might still be required, particularly when considering upcoming recommendations for additional booster dose vaccination for at-risk populations, including healthcare workers [20], due to current and future VOCs. This is of the utmost importance from an occupational health point-of-view: the possibility of reactions causing absenteeism among workers should be taken into account in the planning of workplace vaccinations as well as in the management of affected vaccinees, who should receive appropriate and updated information on the expected reactions of COVID-19 vaccination, reinforcing the mild and transient entity of the majority of events. Indeed, occupational and public health physicians can maintain a fundamental role in providing effective and evidence-based counselling to vaccinees, possibly improving their adherence to national recommendations [21].

The study presents several strengths, including the response rate of around 45% and the sample population of trained medical professionals that could accurately identify and interpret signs and symptoms, potentially reducing under- or over-reporting of specific events and in turn increasing the reliability of the findings. However, the study is limited in a few aspects, such as the study design, with the possible introduction of non-response bias, recall bias, and self-report bias, the limited sample size, as well as the inclusion of a population characterized by uniformity in age and occupational background. In this regard, it must be noted that the survey administration and data collection were performed at different timings after the receipt of the booster dose. This could have introduced further bias in the comparative analysis of the present findings with our previously published results concerning the adverse events following the primary immunization cycle, which by study design were collected in the week following vaccination. Moreover, the assessment of very rare adverse reactions to mRNA vaccines studied in the clinical trials (e.g., allergic reactions, myocarditis, and pericarditis) requires much larger populations. Thus, caution is required in applying these findings to other populations and settings. 

In conclusion, this study adds to our understanding of the type, intensity, and frequency of adverse reactions following an mRNA COVID-19 booster vaccination in young working age categories, an age group that has been shown to be more reactogenic to this vaccine [10]. Nonetheless, more extensive research is required to understand thoroughly the reactogenicity profile of these innovative vaccines, particularly concerning serious and rare events that require the involvement of large populations. The findings of the present study can provide healthcare professionals and policy makers with up-to-date and real-world evidence for appropriate and effective booster vaccination implementation, with the goal of protecting workers and the general public alike.

## Figures and Tables

**Table 1:** Local adverse reactions in the 7 days after the third vaccine dose administration, stratified by symptom severity. “Any” represents the overall reporting of the specific adverse reaction.

	Dose 3 N = 222
Redness, n (% and 95%CI)	
Any	20 (9.0%, 95%CI 5.6–13.6)
Mild	15 (6.8%, 95%CI 3.8–10.9)
Moderate	5 (2.3%, 95%CI 0.7–5.2)
Severe	0 (0)
Grade 4	0 (0)
Swelling, n (% and 95%CI)	
Any	71 (32.0%, 95%CI 25.9–38.6)
Mild	67 (30.2%, 95%CI 24.2–36.7)
Moderate	3 (1.4%, 95%CI 0.3–0.3.9)
Severe	1 (0.5%, 95%CI 0.01–2.5)
Grade 4	0 (0)
Pain at the injection site, n (% and 95%CI)	
Any	196 (88.3%, 95%CI 83.3–92.2)
Mild	113 (50.9%, 95%CI 44.1–57.7)
Moderate	76 (34.2%, 95%CI 28.0–40.9)
Severe	7 (3.2%, 95%CI 1.3–6.4)
Grade 4	0 (0)

**Table 2:** Systemic adverse reactions in the 7 days after the third vaccine dose administration, stratified by symptom severity. “Any” represents the overall reporting of the specific adverse reaction.

	Dose 3 N = 222
Fever, n (% and 95%CI)	
Any	62 (27.9%, 95%CI 22.1–34.3)
From ≥37.5 to 38.0 °C	32 (14.4%, 95%CI 10.1–19.7)
From ≥38.0 to 38.5 °C	24 (10.8%, 95%CI 7.1–15.7)
From ≥38.5 to 39.0 °C	4 (1.8%, 95%CI 0.5–4.6)
From ≥39.0 to 40.0 °C	2 (0.9%, 95%CI 0.1–3.2)
≥40.0 °C	0 (0)
Fatigue, n (% and 95%CI)	
Any	129 (58.1%, 95%CI 51.3–64.7)
Mild	50 (22.5%, 95%CI 17.2–28.6)
Moderate	60 (27.0%, 95%CI 21.3–33.4)
Severe	19 (8.6%, 95%CI 5.2–13.0)
Grade 4	0 (0)
Headache, n (% and 95%CI)	
Any	85 (38.3%, 95%CI 31.9–45.0)
Mild	35 (15.8%, 95%CI 11.2–21.2)
Moderate	43 (19.4%, 95%CI 14.4–25.2)
Severe	7 (3.2%, 95%CI 1.3–6.4)
Grade 4	0 (0)
Gastrointestinal symptoms, n (% and 95%CI)	
Any	7 (3.2%, 95%CI 1.3–6.4)
Mild	5 (2.3%, 95%CI 0.7–5.2)
Moderate	2 (0.9%, 95%CI 0.1–3.2)
Severe	0 (0)
Grade 4	0 (0)
Muscle/Joint pain, n (% and 95%CI)	
Any	98 (44.1%, 95%CI 37.5–50.9)
Mild	53 (23.9%, 95%CI 18.4–30.0)
Moderate	35 (15.8%, 95%CI 11.2–21.2)
Severe	10 (4.5%, 95%CI 2.2–8.1)
Grade 4	0 (0)
Chills, n (% and 95%CI)	
Any	55 (24.8%, 95%CI 19.2–31.0)
Lymph node enlargement, n (% and 95%CI)	
Any	63 (28.4%, 95%CI 22.5–34.8)
Neurological symptoms, n (% and 95%CI)	
Any	3 (1.4%, 95%CI 0.3–3.9)
NSAID/antipyretic use, n (% and 95%CI)	
Any	118 (53.2%, 95%CI 46.4–59.9)

**Table 3:** Prevalence of reporting of one or more moderate adverse reactions interfering with activities following the third vaccination dose, among subjects without severe reactions.

No. of Moderate Reactions.	Dose 3 (222) n, (%)
1	48 (21.6)
2	30 (13.5)
3	12 (5.4)
4	9 (4.1)

**Table 4:** Differences in reporting of systemic adverse reactions and medication use in the 7 days following each of the three vaccine doses administered.

	Dose 1	Dose 2	Dose 3	Cochran’s Q (*p* Value)	McNemar’s Test Dose 1 and 3 (*p* Value)	McNemar’s Test Dose 2 and 3 (*p* Value)
**Redness**	15.3% (95%CI 9.2–23.4)	15.3% (95%CI 9.2–23.4)	8.1% (95%CI 3.8–14.8)	4.129 (0.127)	-	-
**Swelling**	26.1% (95%CI 18.3–35.3)	30.6% (95%CI 22.2–40.1)	34.2% (95%CI 25.5–43.8)	2.837 (0.242)	-	-
**Pain**	95.5% (95%CI 89.8–98.5)	91.0% (95%CI 84.1–95.6)	91.9% (95%CI 85.2–96.2)	2.211 (0.331)	-	-
**Fever**	6.3% (95%CI 2.6–12.6)	29.7% (95%CI 21.4–39.2)	27.9% (95%CI 19.8–37.2)	**31.400** **(0.000)**	**20.571 (0.000)**	0.200 (0.655)
**Fatigue**	41.4% (95%CI 32.2–51.2)	74.8% (95%CI 65.7–82.5)	61.3% (95%CI 51.6–70.4)	**34.066** **(0.000)**	**10.522 (0.001)**	**7.759 (0.005)**
**Chills**	14.4% (95%CI 8.5–22.4)	36.9% (95%CI 28.0–46.6)	27.0% (95%CI 19.0–36.3)	**18.840** **(0.000)**	**6.125 (0.013)**	**3.903 (0.048)**
**Headache**	37.8% (95%CI 28.8–47.5)	52.3% (95%CI 42.6–61.8)	41.4% (95%CI 32.2–51.2)	**7.429** **(0.024)**	0.400 (0.527)	**4.500 (0.034)**
**Muscle/Joint pain**	24.3% (95%CI 16.7–33.4)	55.9% (95%CI 46.1–65.3)	45.0% (95%CI 35.6–54.8)	**29.656** **(0.000)**	**12.302 (0.000)**	**4.500 (0.034)**
**Gastrointestinal symptoms**	5.4% (95%CI 2.0–11.4)	14.4% (95%CI 8.5–22.4)	3.6% (95%CI 1.0–9.0)	**12.400** **(0.002)**	0.500 (0.480)	**10.286 (0.001)**
**Lymph node enlargement**	12.6% (95%CI 7.1–20.3)	22.5 (95%CI 15.1–31.4)	27.9% (95%CI 19.8–37.2)	**11.150** **(0.004)**	**10.704 (0.001)**	1.200 (0.273)
**Neurological symptoms**	0.0%	2.7% (95%CI 0.6–7.7)	2.7% (95%CI 0.6–7.7)	3.600 (0.165)	**-**	**-**
**NSAID/anti-pyretic use**	23.4% (95%CI 15.9–32.4)	55.9% (95%CI 46.1–65.3)	55.0% (95%CI 45.2–64.4)	**37.088** **(0.000)**	**23.113 (0.000)**	0.030 (0.862)

Statistically significant results are highlighted.

## Data Availability

The data that support the findings of this study are available on request from the corresponding author. The data are not publicly available due to privacy restrictions.

## Supplementary Materials

The following supporting information can be downloaded at: https://www.mdpi.com/article/10.3390/vaccines10111779/s1, the Italian electronic form exported from LimeSurvey in a printable format.

## Appendix A

**Table A1:** Classification of the severity of local and systemic adverse reactions following vaccination.

	Mild	Moderate	Severe	Grade 4
**Local reactions**				
**Redness**	From >2.0 to 5.0 cm	From >5.0 to 10.0 cm	>10.0 cm	Necrosis or exfoliative dermatitis
**Swelling**	From >2.0 to 5.0 cm	From >5.0 to 10.0 cm	>10.0 cm	Necrosis
**Pain**	Does not interfere with activity	Some interference with activity	Prevents daily activity	Emergency room visit or hospitalization for severe pain at the injection site.
**Systemic reactions**				
**Fatigue**	Does not interfere with activity	Some interference with activity	Prevents daily activity	Emergency room visit or hospitalization for severe fatigue.
**Headache**	Does not interfere with activity	Some interference with activity	Prevents daily activity	Emergency room visit or hospitalization for severe headache.
**Muscle/Joint pain**	Does not interfere with activity	Some interference with activity	Prevents daily activity	Emergency room visit or hospitalization for severe muscle pain or severe joint pain.
**Gastrointestinal symptoms**	Emesis 1 or 2 times and/or 2 or 3 loose stools in 24 h	Emesis > 2 times and/or 4 or 5 loose stools in 24 h	Requiring intravenous hydration and/or ≥6 loose stools in 24 h	Emergency room visit or hospitalization for severe vomiting and/or diarrhea.

## References

[1] World Health Organization (2021). Statement on the Ninth Meeting of the International Health Regulations (2005) Emergency Committee Regarding the Coronavirus Disease (COVID-19) Pandemic. https://www.who.int/news/item/26-10-2021-statement-on-the-ninth-meeting-of-the-international-health-regulations-(2005)-emergency-committee-regarding-the-coronavirus-disease-(covid-19)-pandemic.

[2] World Health Organization Tracking SARS-CoV-2 Variants. https://www.who.int/activities/tracking-SARS-CoV-2-variants.

[3] Falsey A.R., Frenck R.W., Walsh E.E., Kitchin N., Absalon J., Gurtman A., Lockhart S., Bailey R., Swanson K.A., Xu X. (2021). SARS-CoV-2 Neutralization with BNT162b2 Vaccine Dose 3. N. Engl. J. Med..

[4] Food and Drug Administration (2021). FDA News Release Coronavirus (COVID-19) Update: FDA Takes Additional Actions on the Use of a Booster Dose for COVID-19 Vaccines. https://www.fda.gov/news-events/press-announcements/coronavirus-covid-19-update-fda-takes-additional-actions-use-booster-dose-covid-19-vaccines.

[5] European Medicines Agency (2021). Comirnaty and Spikevax: EMA Recommendations on Extra Doses and Boosters. https://www.ema.europa.eu/en/news/comirnaty-spikevax-ema-recommendations-extra-doses-boosters.

[6] Ministero della Salute Piano Vaccini Anti COVID-19. https://www.salute.gov.it/portale/nuovocoronavirus/dettaglioContenutiNuovoCoronavirus.jsp?lingua=italiano&id=5452&area=nuovoCoronavirus&menu=vuoto.

[7] Niesen M.J.M., Pawlowski C., O’Horo J.C., Challener D.W., Silvert E., Donadio G., Lenehan P.J., Virk A., Swift M.D., Speicher L.L. (2022). Surveillance of Safety of 3 Doses of COVID-19 mRNA Vaccination Using Electronic Health Records. JAMA Netw. Open.

[8] Moreira E.D., Kitchin N., Xu X., Dychter S.S., Lockhart S., Gurtman A., Perez J.L., Zerbini C., Dever M.E., Jennings T.W. (2022). Safety and Efficacy of a Third Dose of BNT162b2 COVID-19 Vaccine. N. Engl. J. Med..

[9] Rahmani A., Dini G., Orsi A., Sticchi L., Bruzzone B., Montecucco A., Pellegrini L., Manca A., Domnich A., Battistini A. (2021). Reactogenicity of BNT162b2 mRNA COVID-19 Vaccine in a Young Working Age Population: A Survey among Medical School Residents, within a Mass Vaccination Campaign, in a Regional Reference Teaching Hospital in Italy. Vaccines.

[10] Lustig Y., Gonen T., Meltzer L., Gilboa M., Indenbaum V., Cohen C., Amit S., Jaber H., Doolman R., Asraf K. (2022). Superior immunogenicity and effectiveness of the third compared to the second BNT162b2 vaccine dose. Nat. Immunol..

[11] Paran Y., Saiag E., Spitzer A., Angel Y., Yakubovsky M., Padova H., Ben-Ami R., Goldinger I., Gamzu R., Sprecher E. (2021). Short-Term Safety of Booster Immunization with BNT162b2 mRNA COVID-19 Vaccine in Healthcare Workers. Open Forum Infect Dis..

[12] Menni C., May A., Polidori L., Louca P., Wolf J., Capdevila J., Hu C., Ourselin S., Steves C.J., Valdes A.M. (2022). COVID-19 vaccine waning and effectiveness and side-effects of boosters: A prospective community study from the ZOE COVID Study. Lancet Infect. Dis..

[13] Naito T., Tsuchida N., Kusunoki S., Kaneko Y., Tobita M., Hori S., Ito S. (2022). Reactogenicity and immunogenicity of BNT162b2 or mRNA-1273 COVID-19 booster vaccinations after two doses of BNT162b2 among healthcare workers in Japan: A prospective observational study. >Expert Rev. Vaccines.

[14] Palm A.E., Henry C. (2019). Remembrance of Things Past: Long-Term B Cell Memory After Infection and Vaccination. Front. Immunol..

[15] Turner J.S., O’Halloran J.A., Kalaidina E., Kim W., Schmitz A.J., Zhou J.Q., Lei T., Thapa M., Chen R.E., Case J.B. (2021). SARS-CoV-2 mRNA vaccines induce persistent human germinal centre responses. Nature.

[16] Cook I.F. (2008). Sexual dimorphism of humoral immunity with human vaccines. Vaccine.

[17] Fish E.N. (2008). The X-files in immunity: Sex-based differences predispose immune responses. Nat. Rev. Immunol..

[18] Paller C.J., Campbell C.M., Edwards R.R., Dobs A.S. (2009). Sex-based differences in pain perception and treatment. Pain Med..

[19] Bauernfeind S., Salzberger B., Hitzenbichler F., Scigala K., Einhauser S., Wagner R., Gessner A., Koestler J., Peterhoff D. (2021). Association between Reactogenicity and Immunogenicity after Vaccination with BNT162b2. Vaccines.

[20] World Health Organization (2022). Interim Recommendations on COVID-19 Vaccination in Autumn 2022 for the WHO European Region: Conclusions and Recommendations of the European Technical Advisory Group of Experts on Immunization. https://www.who.int/europe/publications/i/item/WHO-EURO-2022-5813-45578-65356.

[21] Rief W. (2021). Fear of Adverse Effects and COVID-19 Vaccine Hesitancy: Recommendations of the Treatment Expectation Expert Group. JAMA Health Forum.

