# Supplementary material for: Adverse Reactions after the Third Dose of the BNT162b2 mRNA COVID-19 Vaccine among Medical School Residents in a Regional Reference University Hospital in Italy
Source: Vaccines (Basel). 2022 Oct 22;10(11):1779. doi: 10.3390/vaccines10111779 (PMC9695414; doi:10.3390/vaccines10111779)
Supplement: Supplementary file 1 [file vaccines-10-01779-s001.zip › vaccines-1979119-supplementary.pdf]

LimeSurvey

Questo questionario è finalizzato a indagare le reazioni locali e sistemiche della vaccinazione mRNA COVID-19. Si prega il partecipante di compilare con attenzione il questionario relativo ai 7 giorni a partire da quello della vaccinazione. Questi dati sono molto importanti in ambito di ricerca applicata durante la campagna vaccinale in Italia.

Il questionario può essere salvato, ripreso e modificato fino all'invio da parte del partecipante o alla scadenza prevista.

Sezione A: Sezione 1 - Anagrafica

A1. **Nome e Cognome**

A2. **Genere**

Femminile ☐

Maschile ☐

Non-binario ☐

A3. **Età**

A4. **Quante settimane sono trascorse dalla somministrazione della terza dose?**

A5. **Quanti mesi sono trascorsi tra la seconda e la terza somministrazione di vaccino anti-SARS-CoV-2?**

Sezione B: Sezione 2 - Vaccino COVID-19 mRNA: EFFETTI LOCALI

B1. **Hai avuto arrossamento in sede di iniezione nei 7 giorni successivi alla somministrazione?**

Se AFFERMATIVO, inserire il grado di intensità della reazione (1= Lieve: >2,0 a 5,0 cm; 2= moderato: >5,0 a 10,0 cm; 3= grave: >10,0 cm; 4= grado 4: necrosi o dermatite esfoliativa) e il numero di giorni di persistenza; se NEGATIVO inserire 0 in entrambi i campi.

Grado di intensità ☐

Numero giorni di persistenza

B2. **Hai avuto gonfiore in sede di iniezione nei 7 giorni successivi alla somministrazione?**

Se AFFERMATIVO, inserire il grado di intensità della reazione (1= Lieve: >2,0 a 5,0 cm; 2= moderato: >5,0 a 10,0 cm; 3= grave: >10,0 cm; 4= grado 4: necrosi) e il numero di giorni di persistenza; se NEGATIVO inserire 0 in entrambi i campi.

Grado di intensità

Numero giorni di persistenza

B3. **Hai avuto dolore in sede di iniezione nei 7 giorni successivi alla somministrazione?**

Se AFFERMATIVO, inserire il grado di intensità della reazione (1= Lieve: non interferisce con l'attività; 2= moderato: interferisce con l'attività; 3= grave: impedisce l'attività quotidiana; 4= grado 4: visita al pronto soccorso o ricovero in ospedale per forte dolore al sito di iniezione) e il numero di giorni di persistenza; se NEGATIVO inserire 0 in entrambi i campi.

Numero giorni di persistenza

Grado di intensità

Sezione C: Sezione 2 - Vaccino COVID-19 mRNA: EFFETTI SISTEMICI

C1. **Hai avuto febbre nei 7 giorni successivi alla somministrazione?**

Se AFFERMATIVO, inserire il grado di intensità della reazione (1= ≥37,5-38,0°C; 2= >38,0-38,5°C; 3= >38,5-39,0°C; 4= >39,0-40,0°C; 5= >40.0°C) e il numero di giorni di persistenza; se NEGATIVO inserire 0 in entrambi i campi.

Grado di intensità

Numero giorni di persistenza

C2. **Hai avuto astenia nei 7 giorni successivi alla somministrazione?**

Se AFFERMATIVO, inserire il grado di intensità della reazione (1= Lieve: non interferisce con l'attività; 2= moderato: una limitata interferenza con l'attività; 3= grave: impedisce l'attività quotidiana; 4= grado 4: visita al pronto soccorso o ricovero in ospedale per grave affaticamento) e il numero di giorni di persistenza; se NEGATIVO inserire 0 in entrambi i campi.

Grado di intensità

Numero giorni di persistenza

C3. **Hai avuto brividi nei 7 giorni successivi alla somministrazione?**

Se AFFERMATIVO, inserire il numero di giorni di persistenza; se NEGATIVO inserire 0 nel campo.

Numero giorni di persistenza

C4. **Hai avuto cefalea nei 7 giorni successivi alla somministrazione?**

Se AFFERMATIVO, inserire il grado di intensità della reazione (1= Lieve: non interferisce con l'attività; 2= moderato: una limitata interferenza con l'attività; 3= grave: impedisce l'attività quotidiana; 4= grado 4: visita al pronto soccorso o ricovero in ospedale per forte mal di testa) e il numero di giorni di persistenza; se NEGATIVO inserire 0 in entrambi i campi.

Grado di intensità

Numero giorni di persistenza

C5. **Hai avuto mialgie/artralgie nei 7 giorni successivi alla somministrazione?**

Se AFFERMATIVO, inserire il grado di intensità della reazione (1= Lieve: non interferisce con l'attività; 2= moderato: una limitata interferenza con l'attività; 3= grave: impedisce l'attività quotidiana; 4= grado 4: visita al pronto soccorso o ricovero in ospedale per forte dolore muscolare o forte dolore alle articolazioni) e il numero di giorni di persistenza; se NEGATIVO inserire 0 in entrambi i campi.

Grado di intensità ☐

Numero giorni di persistenza ☐

C6. **Hai avuto sintomi gastrointestinali nei 7 giorni successivi alla somministrazione?**

Se AFFERMATIVO, inserire il grado di intensità della reazione (1= Lieve: vomitare 1 o 2 volte in 24 ore e/o 2 o 3 feci molle in 24 ore; 2= moderato: vomitare >2 volte in 24 ore e/o 4 o 5 feci molle in 24 ore; 3= grave: richiede idratazione endovenosa e/o 6 o più feci molle in 24 ore; 4= grado 4: visita al pronto soccorso o ricovero ospedaliero; se NEGATIVO inserire 0 in entrambi i campi.

Grado di intensità ☐

Numero giorni di persistenza ☐

C7. **Hai utilizzato farmaci antipiretici o antidolorifici nei 7 giorni successivi alla somministrazione?**

Se AFFERMATIVO, inserire il numero di giorni di utilizzo; se NEGATIVO inserire 0 nel campo.

Numero giorni di utilizzo ☐

Sezione D: Sezione 2 - Vaccino COVID-19 mRNA: EVENTI AVVERSI RARI

D1. **Hai avuto linfoadenomegalia nei 7 giorni successivi alla somministrazione?**

Se AFFERMATIVO, inserire il numero di giorni di persistenza; se NEGATIVO inserire 0 nel campo.

Numero giorni di persistenza ☐

D2. **Hai avuto sintomi a carico del sistema nervoso nei 7 giorni successivi alla somministrazione?**

Se AFFERMATIVO, inserire il numero di giorni di persistenza; se NEGATIVO inserire 0 nel campo.

Numero giorni di persistenza ☐

LimeSurvey

Contatti

Servizio di Sorveglianza Sanitaria di Ateneo-Medicina del Lavoro Largo Rosanna Benzi 10, 16132 Genova Padiglione 3 Email: uos.sorveglianzasanitaria2@hsanmartino.it - sorvsanitaria@unige.it

Medici Specialisti e Medici Competenti: Prof. Paolo Durando - durando@unige.it; Dott.ssa Nicoletta Debarbieri - nicoletta.debarbieri@hsanmartino.it; Prof. Guglielmo Dini - guglielmo.dini@unige.it

Servizio di Igiene Largo Rosanna Benzi 10, 16132 Genova Padiglione 3 Email: igiene.segreteria@hsanmartino.it - ambulatorio.vaccinazioni@hsanmartino.it

Medici Specialisti: Prof. Giancarlo Icardi - icardi@unige.it; Dott.ssa Bianca Bruzzone - bianca.bruzzone@hsanmartino.it; Prof. Andrea Orsi - andrea.orsi@unige.it; Prof.ssa Laura Sticchi - laura.sticchi@unige.it

Grazie per aver partecipato all'indagine.
